# Still at School

**Published:** 1872-02

**Authors:** 


### STILL AT SCHOOL.

Some of the members of our profession in a neighboring State (viz.: Kentucky) have recently been taking another lesson in the affairs of professional life, and though they paid the schoolmaster pretty well, they treated him rather roughly in the outcome.

The teacher came to our brethren in the shape of two Knights of the Forceps and Vulcanizer, professing to have instruction of rare value to impart, in which was embraced a process truly wonderful, by which anything having the semblance of an artificial denture, when brought in the vicinity of a mouth, would immediately proceed to locate and attach itself, with such tenacity that nothing less than a flank movement in the dark could in the least induce it to "give down." Upon experiment and demonstration being had, it was found to be "just so"—yes, and it was everlasting, or at least would serve five or six generations. No wonder our brethren became excited about it, and began to devise ways and means for its procurement; it wasn't everybody that could buy that; but perseverance and energy will overcome the greatest difficulties, and in this case all obstacles were surmounted and the thing—whatever it was—was bought and paid for, or at least six hundred dollars' worth of it. The whole stock was not purchased; there was more of the same sort left, to sell at other points.

Now to come down and tell this story in prose: Our brethren in Louisville, Lexington, and several other points in Kentucky, have been most outrageously swindled, by having palmed off upon them a worthless contrivance called "Folsom's patent ridge or edge and flexible border," for artificial dentures. This, like many other worthless things, has an appearance of real value; but it is only in appearance, or at most of very temporary utility —not sufficient to be of a farthing's worth to anybo ly in general practice. By lying and misrepresentation the dentists of Louisville, or some of them, were induced to buy this worthless thing, and not only so, but to give a flattering recommendation to those by whom they had been so grossly deceived.

Learning, very soon after the departure of the impostors, the true nature of the case, officers were sent out upon their track. They were arrested, brought back, and placed in *durance vile*, and subsequently, being brought before a court of justice, it was found that there was no law in Kentucky that would reach the case; which the Judge upon the bench declared was much to be regretted, as law to punish such imposition and swindling was certainly very much needed.

The paper of recommendation was given up and the culprits allowed to go their way with the ill-gotten gain, and the scorn and contempt of every honest man acquainted with the case.

We would suggest that hereafter the profession refuse to purchase any patent, process or instruction, unless known to be of value and have merit.

